# Evaluating the Impact of an Integrated Community Care Model for Older Adults

**DOI:** 10.5334/ijic.9062

**Published:** 2025-11-07

**Authors:** Amanda L. Terry, Leslie Meredith, Jennifer Graham, Eugene Law, Shannon L. Sibbald, Anita Trusler, Amardeep Thind

**Affiliations:** 1 Centre for Studies in Family Medicine, Department of Family Medicine, Department of Epidemiology & Biostatistics, Schulich School of Medicine & Dentistry, Western University, Canada; 2 Centre for Studies in Family Medicine, Department of Family Medicine, Schulich School of Medicine & Dentistry, Western University, Canada; 3 Lambton Public Health, County of Lambton, Canada; 4 Graduate Program in Epidemiology & Biostatistics, Schulich School of Medicine & Dentistry, Western University, Canada; 5 School of Health Studies, Faculty of Health Sciences, Centre for Studies in Family Medicine, Department of Family Medicine, Schulich School of Medicine & Dentistry, Western University, Canada; 6 Department of Epidemiology & Biostatistics, Centre for Studies in Family Medicine, Department of Family Medicine, Schulich School of Medicine & Dentistry, Western University, Canada

**Keywords:** older adults, seniors, age-friendly initiatives, voluntary and community sector, program evaluation

## Abstract

**Introduction:**

The Building an Integrated Community Care Model was a two-year program to support older adults in receiving home and community care services from organizations within the VCS sector in the City of Sarnia and Lambton County, Ontario, Canada.

**Description:**

The ICCM program launched with grant funding from the Ontario Ministry of Health. A goal-based evaluation design was used to assess the implementation and impact of the program. We collected and analyzed data from monthly status reports, surveys of service providers, and client/patient satisfaction surveys, data from key informant interviews, one focus group with service providers, and a reflective discussion.

**Discussion:**

Providers were able to overcome challenges and achieve benefits linked to short-term outcomes. Shared goals amongst providers facilitated the implementation and integration of services. Socially isolated older adults were better served, new partnerships were formed, and community-based initiatives were created. A supportive network of service providers and system planners was created, enhancing the capacity of providers to meet community needs.

**Conclusion:**

This was a complex initiative with multiple organizations coming together in a voluntary governance structure to implement disparate projects. Learnings may be useful to others seeking to implement and assess integrated community care programs for older adults.

## 1. Introduction

### 1.1 Background

An ageing population increases demand on the healthcare system, leading to the need for additional supports from community and health services agencies to enable older adults to age in place [1]. The integration of health and social care services for older adults is one approach to addressing these issues [2,3,4]. Older adults experiencing health disparities and at risk of social isolation are disproportionately impacted by the social determinants of health [5]. Social isolation and loneliness is associated with increased levels of morbidity and mortality [6,7,8,9,10,11], and a greater utilization of health services [12]. Being an older adult [13], living alone [6], and a lack of access to community programs and services [7] are among the risk factors for social isolation and loneliness in the older adult population.

Lambton County is located in Southwestern Ontario, Canada and includes a combination of urban centres (e.g. City of Sarnia), smaller towns (e.g. Town of Petrolia), and rural communities (e.g. Township of Dawn-Euphemia). The County has consistently had a greater proportion of older adults (aged 65 years and older) than the province of Ontario [14]. In response, Lambton Public Health (a public health unit in Southwestern Ontario) identified older adults as a priority, with a commitment to use a healthy aging ‘lens’ to: 1) guide the delivery of public health programs and services; 2) to coordinate with community stakeholders who serve older adults; and 3) to provide leadership in promoting age friendly communities [15]. Age-friendly community initiatives engage multiple interested individuals and organizations across many sectors to ensure that local policies, programs and services are accessible, inclusive and support older adults to age in place [16]. The results from data collection associated with the Age-Friendly Sarnia Action Plan (2017), indicated that older adults in the Sarnia-Lambton community wanted to age in place, that they wanted better information access, and that they had low awareness of services available to support them. Additionally, social isolation and loneliness were identified as risk factors for negative health impacts among older adults; further barriers included limited financial resources and reduced access to health and community services [17]. These findings align with barriers identified in the Canadian context for older adults including: lack of awareness of existing programs and services, insufficient home care services and respite, limited supports promoting older adult independence, lack of health professionals, and financial concerns [18].

### 1.2 Problem Statement

The Voluntary and Community Sector (VCS) has a critical role in the provision of integrated care for older adults. To overcome the barriers and issues identified by older adults, Lambton Public Health led a coalition of individuals and organizations from the VCS to create and implement an integrated community care model. The Building an Integrated Community Care Model (ICCM) was a two year program designed to support older adults in receiving home and community care services in the City of Sarnia and Lambton County. The target audience for the program was older adults who were aging in place, with those individuals experiencing health disparities and those at risk for social isolation as the highest priority. In 2018, the County of Lambton received funding from the Ontario Ministry of Health’s Health and Well-Being Grant Program for the ICCM. The health unit’s healthy ageing strategic priority facilitated partnerships with VCS service organisations supporting older adults. The ICCM program united organizations and service providers from the VCS in an integrated care initiative that can serve as a model, along with others [19,20], for other communities in addressing the needs of older adults. This paper describes the ICCM program and reports on our 18 month evaluation of the program’s activities, implementation, and achievements.

### 1.3 Description of Program

The ICCM program had three main objectives: 1) to improve linkages between local health and social sectors with an online referral network; 2) to improve information access for older adults, their families and their caregivers; and, 3) to increase capacity for community outreach services. Activities associated with the first two objectives included: 1) building a referral network for Sarnia-Lambton health care and social services sectors; and, 2) creating a user-friendly interface for the Healthline website, which served to increase the connections among community service agencies and the people of Sarnia-Lambton. A situational assessment was conducted for the Healthline website to inventory health and social services available for older adults. For the third objective, three programs were expanded: 1) the pilot Community Paramedicine Outreach program; 2) Adult Day Program; and, 3) Tel-Check program. These expansions offered additional outreach services to older adults in need due to health disparities or social isolation. The Community Paramedicine program and the Adult Day Program, the Community that Cares project, as well as the user-friendly interface project received funding during the first year of the two-year grant period. The Referral Network and Tel-Check projects were funded for both years. See Table 1 for an overview of the ICCM program components. The ICCM program implementation was led by Lambton Public Health and was guided by the Community Support and Health Services Sub-Committee, which is part of the larger Age-Friendly Sarnia initiative. The sub-committee comprised community partners representing a range of service providers, older adult groups, and policy makers in Sarnia-Lambton.

**Table 1:** Elements of the ICCM Program.


ICCM PROGRAM ACTIVITIES	IMPLEMENTATION ACTIONS	PURPOSE	ORGANIZATION/PROGRAM RESPONSIBLE FOR IMPLEMENTATION

Create an On-Line Referral Network	Procured and implemented a software platform to manage referrals	Improve linkages between local health and social sectors via a referral network, where clients could be referred from one provider agency to another.	Lambton Elderly Outreach and Lambton Public Health

Conduct Situational Assessment	Review and collation of community and health support services	To provide accurate and complete information for the Healthline.ca website.	Lambton Public Health

Develop a User-Friendly Interface for the HealthLine.ca Website	Create a user-friendly interface and test the interface with community members	Enhance the design of the Healthline.ca website to increase the connections among community service agencies and the residents of Sarnia-Lambton.	Lambton Public Health/Erie St. Clair Local Health Integration Network and the City of Sarnia partner organizations

Increase Capacity of Tel-Check Program	The Tel-Check program added a new staff member to assist with program promotion and volunteer recruitment and training.	Support socially isolated older adults via a service that provided a daily telephone call to older adults and persons with disabilities for the purpose of social interaction and medication reminders.	Family Counselling Centre/Tel Check Program

Expand Community Paramedicine Program	The Community Paramedicine Project: 1) held Wellness Clinics that were open to the general public throughout Lambton County, and 2) increased their capacity to deliver home visits to clients referred by community providers.	Provide outreach services to high-risk older adults (high users of emergency medical services and the health system) through home visits and Wellness Clinics to promote aging in place/independent living.	County of Lambton Emergency Medical Services/Community Paramedicine Program

Expand Lambton County Adult Day Program	Increased the Registered Practical Nurse (RPN) staffing hours from part time to full time in the Adult Day Program, thereby enhancing capacity to complete health monitoring assessments.	Increase health care monitoring services for clients of the Adult Day Program & client referrals to other services. The Adult Day Program provides individualized support to older adults living in the community and their caregivers.	County of Lambton Long Term Care/Adult Day Program

Implement a Community that Cares Campaign	With the assistance of the Community Support and Health Services Sub-committee, host events (e.g. workshops and fairs) and create products (e.g. videos) and promotional resources (e.g. pens).	Promote available supports and volunteer capacity needs. Inspire individuals, groups, and organizations to volunteer and to support causes they cared about.	Lambton Public Health


**Figure 1:**
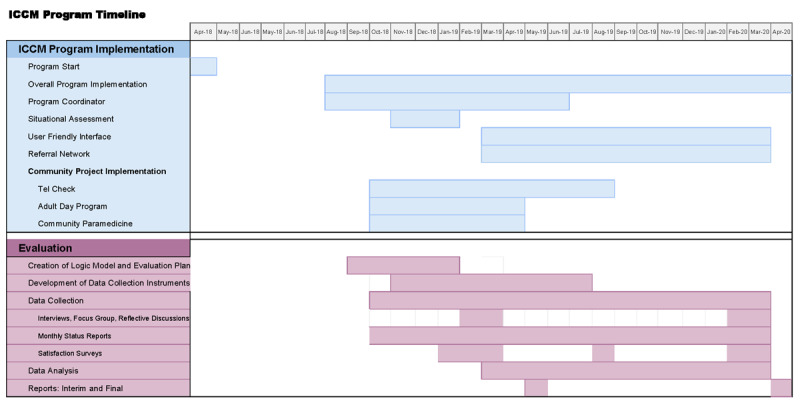
ICCM Program Implementation and Evaluation Timeline.

**Figure 2:**
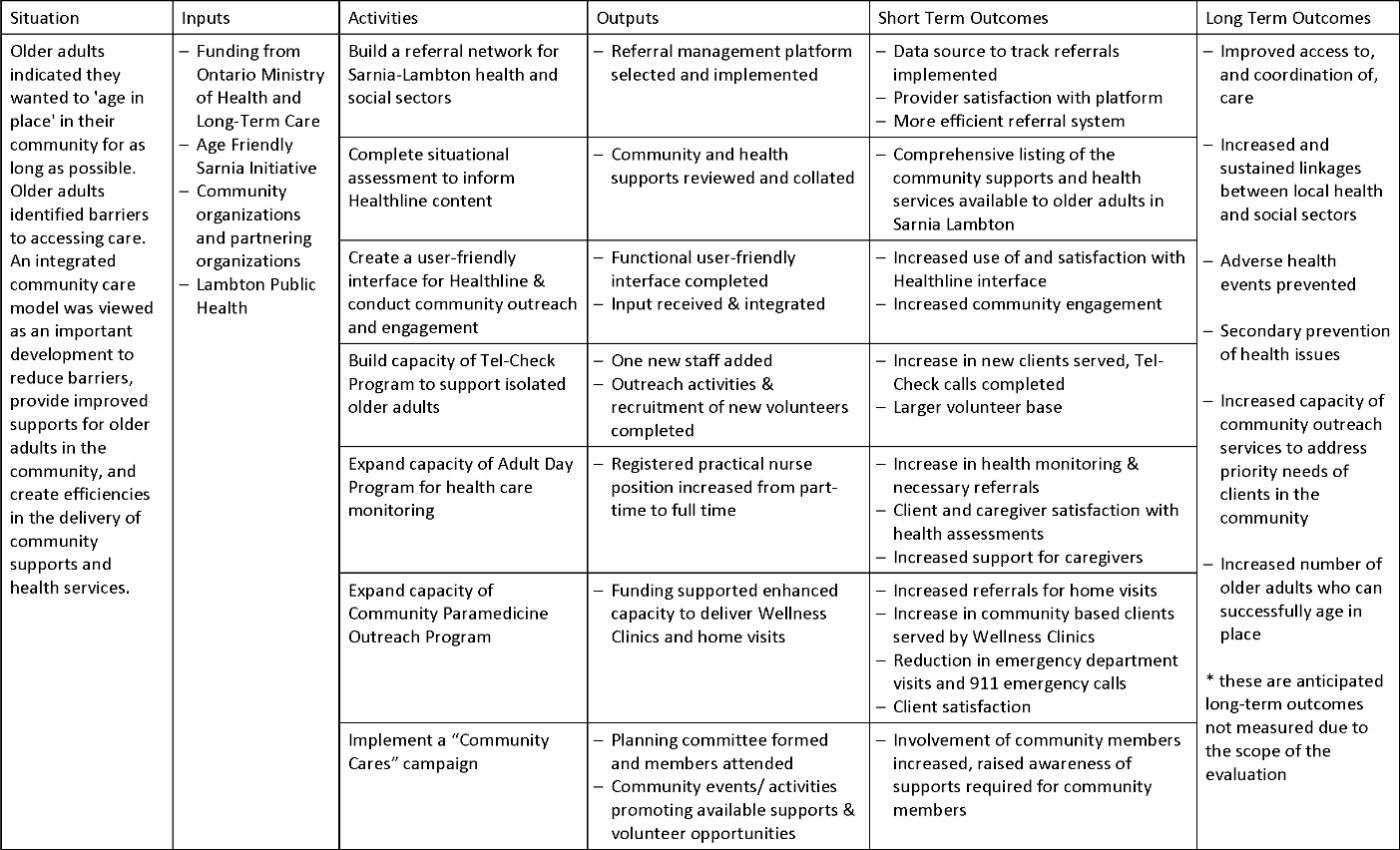
Logic Model Overview – Building an Integrated Community Care Model for Sarnia Lambton.

Please see Figure 1 for an overall timeline of the ICCM program implementation and evaluation activities. Part of the evaluation activities included the development of a logic model which functioned as a framework that guided the overall evaluation (see Figures 1 and 2).

### 1.4 Involvement of People with Lived Experience

People with lived experience, including two older adults, were part of the Age-Friendly Lambton Steering Committee and also actively advised the ICCM program. Members of the Sarnia-Lambton community, including older adult groups, were extensively involved in the activities of the ICCM program (See section 3.0).

## 2. Evaluation Methods

### 2.1 Study Design

Given the complexity of the ICCM program and its implementation, including multiple organizations from the VCS, disparate projects, specific client groups, and the voluntary nature of its governance structure, we used a goal-based evaluation design to assess the implementation of the ICCM program, the achievements associated with the program’s objectives, and to report on short term outcomes [21]. In consultation with Lambton Public Health, a logic model (see Figure 2) was created based on the activities proposed for the ICCM program (see Table 1) describing the anticipated inputs, activities, outputs and outcomes of the project; this functioned as a framework to guide the overall evaluation. The design included a mixed methods approach to data collection and analysis, intended to qualitatively and quantitatively appraise the extent of the implementation of the project and its overall impact.

### 2.2 Indicators, Data Collection and Analysis – ICCM Program

The following indicators (see Table 2) were used to assess the process of the ICCM program implementation, as well as the program’s outputs and short term outcomes. Data collection activities for these indicators utilized both quantitative and qualitative methods.

**Table 2:** Activities, Indicators and Data Collection Methods.


ACTIVITY	INDICATORS	DATA COLLECTION	

		QUANTITATIVE	QUALITATIVE

1. Build a referral network for Sarnia-Lambton health and social sectors	Number of service agencies in the referral management network # of referrals # of service providers who completed training to navigate the new system Satisfaction levels of users	Monthly status reports; satisfaction surveys	Interviews

2. Complete situational assessment to inform Healthline content	# of organizations from which data were collected Partner input received	Analyze existing programs	Reflective Discussion

3. Create a user-friendly interface for Healthline & conduct community outreach and engagement	Interface created # and type of outreach activities # of people engaged through outreach activities # of hits/searches on new information interface Satisfaction levels of users	Monthly status reports; satisfaction surveys	Interviews; program/meeting notes

4. Build capacity of Tel-Check Program to support isolated older adults	# of new clients enrolled # of volunteers recruited and trained # of calls made # of clients referred to the program # of clients receiving medication reminders	Monthly status reports; satisfaction surveys	Interviews; reflective discussion

5. Expand capacity of Adult Day Program for health care monitoring	# of health monitoring assessments completed # of health risks/issues identified for clients and caregivers Satisfaction levels of clients and caregivers	Monthly status reports; satisfaction surveys	Interviews; reflective discussion

6. Expand capacity of Community Paramedicine Outreach Program	# of clients in home visit program # of referrals to other community agencies 911 calls and ED visits (home care clients) # of clients served at Wellness Clinics # of Wellness Clinics Patient satisfaction (home visits and wellness clinics)	Monthly status reports; satisfaction surveys	Interviews; reflective discussion

7. Implement a “Community Cares” campaign	# of planning committee meetings and attendance # of community partners involved # of community events and participants # of promotional resources distributed in community # of products created (e.g., logo, impact stories, concept maps)	Monthly status reports; satisfaction surveys	Interviews; reflective discussion; program/meeting notes


#### 2.2.1 Quantitative Data Collection and Analysis

The situational assessment produced an inventory of health and social services in Sarnia-Lambton available for older adults. Information on services was extracted into an excel spreadsheet. Tailored status reports were created to capture indicator information from each service provider on a monthly basis. These reports were reviewed and collated by evaluation team members, serving as a check point to assess their quality. Satisfaction questionnaires were completed by clients of the Community Paramedicine Program, Adult Day Program and Tel-Check program; these data were summarized and shared with the evaluation team. The questionnaires also contained open-ended questions, from which the quotes reported in the results section are drawn. A survey was conducted with the users of the referral network to assess its helpfulness, efficiency, and to assess levels of satisfaction. Number of referrals was collected in Year 2. A questionnaire was administered via the Age-Friendly Sarnia website to assess satisfaction with the user-friendly interface. All quantitative data were collated and summarized by the evaluation team.

#### 2.2.2 Qualitative Data Collection and Analysis

Interviews were conducted with eleven individuals from the Tel-Check Program, Adult Day Program, Community Paramedicine Program, and the referral network; a focus group was conducted with providers from the Community Paramedicine Program. This data collection was repeated at the end of each program implementation year. The interviews and the focus group were digitally recorded and transcribed verbatim. The team took a descriptive qualitative approach to the analysis of the data [22,23]. Evaluation team members individually reviewed initial interview/focus group transcripts, noting themes and important quotes. Transcripts were reviewed as a team and we noted collective themes. Each team member then individually reviewed the subsequent transcripts with the identified themes in mind, iteratively adding new themes and identifying quotes. A final team meeting was conducted to consolidate the themes across all interviews/focus group. All ICCM service providers were also brought together by the evaluation team to discuss how the ICCM program was progressing. Information from this session was then reviewed and summarized. These qualitative data were particularly helpful in understanding the implementation of the ICCM program.

These are program evaluation activities and therefore are considered exempt from human ethics review in accordance with Article 2.5 of the Tri-Council Policy Statement: Ethical Conduct for Research Involving Humans [24].

## 3. Results

In this section, we report on the evaluation results in relation to the outputs and short-term outcomes for the activities associated with the three main objectives of the ICCM program.

### 3.1 Quantitative Results

Activity 1: Build a referral network for Sarnia-Lambton health and social sectors

An on-line referral management platform for home care and community service providers was selected and implemented. Fifteen local agencies were recruited with 10 ultimately agreeing to participate. The referral network had 115 services listed. In year 2 of the ICCM program, 181 referrals were received and 184 were sent out. Individualized training was provided to 7 agencies as well as 2 informational/training webinars for all 10 agencies. Twelve individuals responded to the satisfaction survey about the referral network. Although most found the system helpful overall, those receiving referrals found it less helpful (5 of 9 responses expressed that the system was not helpful). Many of the open-ended comments centred on the fact that key agencies (larger organizations who often make referrals) were not using the system. As described below in section 3.2.2 the referral network was not taken up by the originally planned number of service providers, because of the uncertainty surrounding the Local Health Integration Networks (the main referral partner) and the reluctance of large local organizations to make a change in referral software and processes. Additionally, funding delays and a change in the lead organization significantly delayed the implementation of the referral network.

Activity 2: Complete situational assessment to inform Healthline.ca content

The situational assessment identified and collected information on 133 organizations providing community supports and health services to older adults in the community. The results of this assessment informed the content for the Healthline website and the user-friendly interface.

Activity 3: Create a user-friendly interface for Healthline.ca & conduct community outreach and engagement

Building on the results of Activity 2, the user friendly interface for the Healthline website was created and launched. User testing was completed by members of the Community Support and Health Services sub-committee. There were 27 outreach and education events with community partners to gather feedback on the interface design and content as well as to provide information. In response to feedback from the community, a print version of the website was created; 3200 Senior’s Guide booklets were distributed to 24 community agencies as well as at community events. Over the 10-month implementation period of the Healthline website there were 4775 website users with 89% (4434) of users not having visited the site before. Thirty-three individuals responded to the user satisfaction questionnaire; over half were service providers with the others being caregivers, older adults or other clients looking for services and volunteers. Thus, the website was able to reach its target audience. Seventy-seven percent of respondents said they found what they were looking for which included transportation, help at home, services for the disabled, dementia supports, leisure classes, opportunities to volunteer, and housing support among others. Eighty two percent of respondents were either satisfied or very satisfied with the ease of using the website, with 3% expressing dissatisfaction.

Activity 4: Build capacity for the Tel-Check program to be able to support socially isolated older adults. [funded for both years of implementation]

The Tel-Check program added a new staff member to assist with program promotion and volunteer recruitment and training. This program received referrals for 87 clients from local agencies, added 68 new clients with 24 of these clients receiving medication reminders, and made 11,521 phone calls. Calls per month increased and calls more than doubled from year 1 (3483) to year 2 (8038). The program recruited 381 volunteers, of which 57 received program training. This training was provided so that the volunteers would be prepared for their interactions with clients during their phone calls for social exchange and medication reminders. The staff member increased marketing and outreach activities to reach volunteers (711 activities) and providers who could refer clients (84 activities). Posters and brochures were distributed at many locations e.g. libraries. Public service announcements were made on local radio stations. A Tel-Check employee attended local events to promote the service. These activities resulted in an increased volunteer base and capacity to deliver services for the Tel-Check organization. A Tel-Check client noted the following about the service: “Receiving Tel-Check calls puts me in a better mental space. Don’t know what I would do without your service. It is lifesaving at times”.

Activity 5: Expand capacity of Adult Day Program for health care monitoring [funded for year 1 of implementation]

The Registered Practical Nurse (RPN) staffing hours were increased from from part time to full time in the Adult Day Program, thereby enhancing the program’s capacity to complete health monitoring assessments. There were 471 health monitoring assessments completed which included: cognitive health testing, and physical health testing (e.g. vital signs). Of the 207 assessments completed in one-year, 8 health risk/issues were identified. There were 216 referrals made to address client/caregiver needs, including to other community programs focusing on seniors (e.g. Alzheimer’s Society), transportation, family doctor, home service programs and home maintenance services. Additional questions around health monitoring/services were added to the regular satisfaction survey of the Adult Day Program for the ICCM Program. The results showed that clients and caregivers were satisfied with health assessments (96% satisfied or very satisfied), found them beneficial (96%) and wanted them to continue (96%). The caregiver for a client stated the following regarding health assessments: “I think that it is good that information is shared and updated regularly so that everyone is aware of any health issues or concerns”.

Activity 6: Expand capacity of Community Paramedicine Outreach Project [funded for year 1 of implementation]

The Community Paramedicine Outreach project was funded by the ICCM program for the first year of the grant. The project was able to continue for a second year with other sources of funding; data were also collected in the second year. There were 63 new client referrals to the home visiting program. Home visits were provided by community paramedics in a client’s home and included identification of health issues, referrals, and teaching such as how to cope with pain. Community paramedics operate in an expanded role that requires additional training. Data for 911 calls and emergency department visits were only available for Year 1 and are based on 14 new home visit clients. Among these clients, emergency department visits decreased 78% (from 6 months prior to admission to the home visit program) and 911 calls for those clients also decreased from 18 calls in one year prior to enrollment to 9 calls in one year post enrollment. The community paramedics also referred 29 clients to other community resources during the two-year timeframe. One hundred and thirteen Wellness Clinics, delivered by community paramedics, served 1,270 clients, with an almost twofold increase from year 1 to year 2. Wellness clinics, which included vital sign checks, flu shot administration, answering health and wellness questions as well as connecting people with community services as needed, were held throughout Lambton County to reach those most in need of community-based care. Locations included older adult apartment complexes, arenas/sports complex, recreation centres and churches. In Year 1, satisfaction surveys were conducted with home visit clients and wellness clinic clients. Of the 1,270 Wellness clinic clients, 43 completed questionnaires; seven home visit questionnaires were completed. The results showed that all clients were very satisfied with the care provided and agreed that the Community Paramedicine home visits helped them remain in their home safely and independently. Wellness clinic clients indicated that they were ‘very satisfied’ (84%) with care provided, that their needs were met (100%), and that their knowledge of their own health (97%) and preparation to deal with future health care concerns (100%) improved. When asked about the overall benefits of the Wellness clinics, one client noted, *“I am able to receive advice and make plans to better my health”*. A similar sentiment was expressed by a home visit client *“[I] receive immediate attention and I learn more and more about my condition”*.

Activity 7: Implement a “Community Cares” campaign.

The Community that Cares campaign was funded for the first year of the program. The purpose of this campaign was to increase the involvement of community members in planning and promoting the supports available for older adults, and to raise awareness of the need and opportunities for volunteers to help older adults age in place. The project was led centrally by Lambton Public Health, with substantial planning and implementation guidance from the Community Support and Health Services Sub-Committee. Twenty-seven community partners met to work on the campaign. This group had 45 meetings that attracted 312 people. The group was able to hold or participate in seven events, for example, Healthy Communities workshops, and a Housing Fair that attracted 402 active participants. The group was also able to create 60 products, e.g. a logo, banner, community partner videos, website, video impact stories, newsletters and news articles. There were 3,211 promotional resources created and distributed to the community (e.g. water bottles, mugs).

### 3.2 Qualitative Findings

Implementation of the ICCM Program was facilitated by the presence of a shared goal amongst service providers to support older adults living in the community to age in place, and by the existing Community Support and Health Services Sub-Committee structure. Themes identified from the qualitative data related to facilitators, challenges and other outcomes.

All quotes appearing in the text are directly from project participants.

#### 3.2.1 Facilitators

Six main themes were identified which represent facilitators of the implementation of the service expansion.

**Match Between Community Needs and Project Objectives**

Each activity began with objectives for meeting a need in the community. For example, the User Friendly Interface was identified as a community need through a process of community engagement which focused on what was needed in the community to be age friendly.

*“…the user friendly interface was created in part from our action plan. So it was a need in the community that was discovered… for that first outcome we definitely created a functional interface that connected with our original action plan.”* Service provider

**Existing Services & Structures**

Implementation of ICCM was facilitated by building on services that already existed e.g. Healthline website. The structure provided by the Community Support and Health Services Sub-Committee (i.e., regular meetings, reporting requirements) and its leaders helped to keep activities on track.

*“…what was nice is…the work of this grant was born out the Community Support and Health Services Subcommittee of Age Friendly Sarnia. So we already had a group at the table.”* Service provider

**Partnerships/Collaborations**

Participants understood the importance of building relationships and creating and maintaining partnerships to produce successful outcomes. They valued the opportunity to interact and collaborate with other service providers in Sarnia-Lambton.

*“The committees that… have been moving forward created a new kind of interconnectedness with other agencies who are providing supports and services for seniors… that’s been a really good component to this as well, because it just enhances our knowledge-base and helps to build collaboration”*. Service Provider

*“…an exciting opportunity [to share volunteers] that came to us … it just evolved out the communications that wouldn’t have happened without this [ICCM] project”* Service Provider

**Marketing/Promotion/Awareness**

Marketing and promotion were perceived as essential facilitators to raising awareness of the services offered and to ensure increased use of these services. Providers increased their understanding of the services available in Sarnia-Lambton and how those involved in ICCM initiative could cross- promote and leverage each other’s services. The continued, ongoing promotion of the services was viewed as essential to the continued success of the ICCM.

*“[As a result of this program] there’s been a greater impact than there has been in years for this community in terms of getting buy in from the community… heightening their awareness on the services that are out there…the challenges that seniors are facing…[and] of how people can get involved.”* Service Provider

**Leadership**

Many important facets of leaders and leadership emerged. ICCM participants felt it was important to build trust within the community. There was universal praise for the leader of the ICCM Program. Efforts in reaching out to potential partners, reinforcing the positive achievements that had been achieved, and pursuing new opportunities as they arose, were viewed as facilitators of success. The time commitment and effort necessary to develop the partnerships required to create, implement and successfully complete the ICCM program required dedication and strong leadership.

*“I think it’s the energy and enthusiasm and that inherent desire to keep moving forward…and to make our community accessible to all, I think we all buy in to that”* Service Provider

**Adaptation**

As with any new initiative, some projects could not be implemented exactly as planned, which required adaptation and flexibility. As an example, the user-friendly interface project was intended to be a digital-only platform, however a printed version of the information was created when it became clear from community engagement activities that a printed guide was desired.

*“…whatever we need to do we have the flexibility and ability to kind of address those specific needs or that specific culture that’s going on at the time*.” Service Provider

#### 3.2.2 Challenges

Three themes represent the challenges that service providers faced in their implementation of service expansion and changes:

**Time**

The challenge posted by time constraints repeatedly emerged, this included: decreased time to complete projects, the need for dedicated staff time to complete the implementation, and the time lag for decisions to be made when decision makers were unavailable. Service providers found it challenging to devote the time necessary to maintain partnerships and collaborations, including attending and participating in committee meetings on a regular basis, yet most fulfilled this commitment

*“In that short amount of time, we did do a lot.”* Service Provider

**Organizational Roadblocks/Processes**

There were instances when project implementation was impeded by organizational processes. To ensure uptake, key players needed to be on board early and actively involved; this did not happen within all the ICCM activities. While those directly involved with implementing the ICCM program were fully committed, there were situations where organizational leadership did not support service promotion, and there was resistance to change. Other challenges included providers having overlapping services that they therefore needed to coordinate. A final but important challenge was the lack of a full-time coordinator for the second year of the ICCM Program, as there was only funding for this position for the first year of the program.

“*There was opposition from [organization name] here full stop…their [leader] wanted nothing to do with our program.”* Service Provider

“*…even though we’re trying to support the needs of similar individuals in our community there’s a lot of competing priorities and competing mandates and then that made it kind of difficult to move forward*.” Service Provider

**Contextual Changes**

The challenges posed by the change and uncertainty in the health system during the time frame of ICCM Program cannot be understated. Health system transformation continued throughout the two years of the program’s implementation. The state of uncertainty surrounding the Erie St. Clair Local Health Integration Network’s participation in the program led to a cascade of consequences including delaying the start of the project and hindering the participation of the largest agency in the referral network. This led to a reluctance on the part of other service providers to join the network, which then held other agencies back from participating fully.

*“Definitely health system transformation was a challenge for us”* Service Provider

*“I feel there are not enough community partners using this service [referral network] and the Local Health Integration Network is crucial to making this program successful.”* Satisfaction Survey Respondent

##### Other Outcomes

Many of the service providers were able to consult the community and incorporate those ideas into the design and implementation of their services, for example the creation of a user-friendly interface for the Healthline information service incorporated significant community engagement for testing and providing feedback. In another example, the Community Paramedics sought to add immunizations to the Wellness Clinics based on community-expressed needs.

*“…I’ve seen that this grant brought more agencies together and remained active and brought community members together too, to be part of it and have input”* Service Provider

*“I think it’s very important that we are not doing ‘for’ the community, that we are doing ‘with’.”* Service Provider

## 4. Discussion

The goal of the evaluation was to assess the ICCM program’s implementation, and the achievements associated with program’s objectives. The steps taken to implement the ICCM Program, including the identification of community needs, developing partnerships, having shared goals to support collaboration, targeting resources, and building capacity, are consistent with the guidance provided by the World Health Organization age-friendly initiatives [25]. Many of the barriers and facilitators to the program’s implementation echo those that have been previously identified in age-friendly community initiatives [26,27,28]. The ICCM Program had characteristics such as the breadth of organizations and individuals involved from the VCS, and projects with different mandates, that differ from other examples of integrated community care models [19,20]. Also, integrated care approaches for older adults more commonly focus on clinical care coordination [4] versus a broader, multi-sectoral approach that includes VCS in these models [2]. With respect to the early stages of the ICCM program, the focus was on co-operation and collaboration. This meant building connections amongst the various partners. This is similar to the linkage or early co-ordination level of care integration [29]. In these early stages, effort was focused on developing trust, opening clear lines of communication, and finding ways to work together more effectively [30]. These early relationships and shared practices laid the groundwork for moving toward stronger integration, and a focus on governance, resources, and service delivery.

Overall, the implementation was successful and led to positive impacts in several areas, including those specific to the project objectives, and those that were more broadly applicable. Specific benefits centre around the connectedness of the community at the public, client and provider level. A referral network is now in place that can be used to facilitate referrals among agencies. A user-friendly interface that assists those in the community to learn about services available and contact services of interest was created. Other benefits include increased call capacity and volunteer recruitment within the Tel-Check program; Wellness Clinics that provided access to health checks; and, the increased capacity of the Adult Day Program to conduct health assessments and provide caregiver support. The program team also ran a very successful “Community that Cares” campaign. Administratively, the grant was very successful. Several aspects of the ICCM supported the implementation of all programs and the initiation of new collaborations and partnerships, these included: 1) the leader of the ICCM Program; 2) the support staff (program coordinator role); and 3) the structure created by leveraging the existing Community Support and Health Services Sub-Committee of the Age Friendly Sarnia initiative. ICCM Program participants’ experiences were universally described as positive with an emphasis on the opportunities provided, knowledge gained, connections made, and lessons learned. Two overarching benefits of the program that were not directly tied to the original objectives were: 1) the development of a supportive network of service providers and interested individuals within the VCS; and 2) the enhanced capacity of service providers to meet community needs because of the learning they experienced through the program. The presence of a shared goal amongst providers to support older adults living in the community to age in place, and the existing committee structure for the project facilitated the development of a true network of service providers and stakeholders.

The project was not without its challenges. Issues of delayed funding and health system change influenced the overall program. These issues also resulted in challenges in four areas.

First, it was difficult to implement and promote the uptake of a new referral system within the available time frame. Providers were wary of the work involved in implementing the system and were concerned about its sustainability once the grant ended. Although the referral network was implemented, the uptake was not ideal. This includes the number of agencies who signed on, as well as the number of referrals being made through the system. All efforts were made by the program team to increase participation but the health system transformation underway at the time hindered the project in reaching its full potential.

Second, the program coordinator position was funded for a one-year period. Ideally, the coordinator would have functioned over the two years of the grant to facilitate the full implementation of the program. Lambton Public Health provided in-kind support to assist in completing the grant requirements over the last few months of the program.

Third, ongoing health system transformation was a definite challenge for the ICCM Program as a whole, but more specifically for Lambton Public Health, the referral network project and the Community Paramedicine project. Both public health and the paramedic services were identified as parts of Ontario’s health care system that would be reorganized and amalgamated but no details or timelines were available, creating uncertainty about how the transformation would unfold. Local health system planning organizations were targeted for closure or at least substantial reorganization and were therefore unable to follow through on their initial commitments to ICCM program, which included leading both the referral network and the user-friendly interface projects. The interface was able to recover, with other organization stepping up to implement the project with no real consequence other than a time delay.

Fourth, the length of the grant period hindered the ICCM Program’s ability to realize its full impact and achieve longer-term outcomes. Many of the projects were provided one year of funding; however, even two years of funding is a short time frame for successful project implementation. Three of the projects created a new product or service. A new service like the Wellness Clinics needs time to promote and ‘spread the word’ regarding its existence to the communities involved, and to set the expectations of the service to be delivered. There is usually a slow start and then steady increases as this type of service matures. Although these projects did incredibly well to create and implement new services, a longer implementation period would have assisted them in reaching their full potential and demonstrating longer-term impact.

Pertinent to this discussion are two scoping reviews, one published in 2023 by Hong et al. [27], and one published in 2024 by Forsyth and Lyu [28], as well as an interpretive review published in 2022 by Menec and Brown [26] that offer a comprehensive overview of the state of the literature with respect to the factors that can facilitate or impede the success of age-friendly initiatives to support community-dwelling older adults. Many of these factors are consistent with the findings of this evaluation, and include: 1) the presence of strong and diverse partnerships among individuals and organizations from the VCS [26,27]; 2) a local project champion (ICCM leader) [26,28]; 3) structures that utilize existing resources such as the Community Support and Health Services Sub-Committee [26,28]; 4) a specific staff role to support the initiative (ICCM program coordinator) [26]; 5) program funding [26,28]; and 6) leadership across levels and a strong vision [26]. Several of the steps that the ICCM program undertook, including the “Community that Cares” campaign, have been identified as important strategies to raise awareness and gain support for age-friendly initiatives [26,28]. An important outcome of the project was the connectivity and sense of community that the ICCM Program participants developed [28].

## 5. Lessons Learned

Key elements of sustainability were included in the grant proposal and implementation plans for the ICCM Program. This was critical to the success of the program and its ongoing sustainability.

The following five points are lessons learned regarding the future direction of the ICCM Program:

- **Build Awareness and Promote Services**
  To serve the maximum number of clients, and involve as many community partners as possible, it will be important to continue to share the program’s services and achievements with funders, policy makers, providers and the community at large. Activities to enhance awareness of age-friendly initiatives, to promote them, and strategies to gain public support can support the successful implementation of age-friendly programs [26].
- **Support for the Program**
  To ensure ongoing sustainability of the program and to continue to realize its associated benefits, it will be important to secure funding through external grant competitions or by the incorporation of program elements within existing service organizations, as these factors can facilitate the ongoing nature of age-friendly initiatives [26]. In keeping with this, building in time for staff to participate in data collection, attend meetings, and interact with the community would also support the program’s continuation and its ongoing success.
- **Build on existing structures**
  A great deal of success of the ICCM Program was fostered by the structural elements that supported the overall program, and the strong link with existing community needs. As much as possible, it will be important to ensure that all major players in the health system are engaged when moving forward with program sustainability. Continuing with the structure and strong leadership of the existing committees, and convening ad hoc meetings to address specific needs, will serve to foster the ongoing success of the program. Successful age-friendly initiatives include these structural elements [31,26]. Building on the existing collaboration and partnerships that are part of the ICCM Program will also support the move toward integrated community care provision.
- **Collect and Report Data on Outcomes and Indicators of Success**
  It will be important to continue to collect data on appropriate, measurable indicators for each individual project including longer term outcomes for the initially promising impact shown by the Community Paramedicine Outreach project, the Adult Day Program, and the Tel-Check program. More emphasis should be placed on collecting qualitative data from the clients of the programs to understand the impact of the service provision. As well, tracking meetings, new collaborations and other relevant indicators should be collected at the ICCM Program level. These data should be reported on to funders, policy makers, other service providers and the community. This step follows the guidance offered by the World Health Organization to communities that are seeking to become age-friendly [25].

## 6. Limitations

Due to the timing of the grant proposal submission for the ICCM program, the evaluation team was constrained in terms of the evaluation design. Limited resources were another handicap. As a result, the final design emerged as a process evaluation with a post-test only design. An outcome evaluation with a pre-test post-test design would have been a preferable design, and a pre-test post-test with a control group (e.g. a neighboring County as a control) would have been ideal. However, as mentioned above, resource and time constraints precluded these designs. In addition, data collection was not fully under the evaluation team’s control as we relied on quantitative data submitted by the service providers to assess the program’s implementation and achievement of short-term outcomes. Due to the evaluation time frame, we were only able to observe the process implementation and short-term outcomes associated with the ICCM Program, leaving long-term outcomes and sustainability unmeasured.

## 7. Conclusion

Quantitative and qualitative evaluation findings indicate mixed success in the implementation of the ICCM program. There were contextual and implementation challenges with the project that led to delays in implementation, particularly with the referral network. However, socially isolated older adults were served and were satisfied, partnerships were formed, and community stakeholders were consulted and included in community-based initiatives. New and expanded services were provided. Although there were system, program, and project level challenges, the service providers were able to persevere and implement the planned projects. This was a complex initiative with multiple organizations coming together in a voluntary governance structure to implement disparate projects. Learnings may be useful to others seeking to implement and assess integrated community care programs for older adults.

## References

[1] Carter C, Iciaszczyk N, Sinha S. Health Care Access Among Older Canadians: Findings from the NIA’s Ageing in Canada Survey. National Institute on Ageing. Accessed November 29. 2024. https://www.niageing.ca/access-to-health-care-2024.

[2] Dambha-Miller H, Simpson G, Hobson L, et al. Integrated primary care and social services for older adults with multimorbidity in England: a scoping review. BMC Geriatr. 2021;21(1):674. DOI: 10.1186/s12877-021-02618-8 34861831 PMC8642958

[3] Wodchis WP, Dixon A, Anderson GM, Goodwin N. Integrating care for older people with complex needs: key insights and lessons from a seven-country cross-case analysis. Int J Integr Care. 2015;15(6). DOI: 10.5334/ijic.2249 PMC4628509 26528096

[4] Briggs AM, Valentijn PP, Thiyagarajan JA, Araujo De Carvalho I. Elements of integrated care approaches for older people: a review of reviews. BMJ Open. 2018;8(4):e021194. DOI: 10.1136/bmjopen-2017-021194 PMC5892746 29627819

[5] Government of Canada, Employment and Social Development Canada. Social isolation of seniors – Volume 1: Understanding the issue and finding solutions. February 22, 2017. Accessed November 29, 2024. https://www.canada.ca/en/employment-social-development/corporate/seniors-forum-federal-provincial-territorial/social-isolation-toolkit-vol1.html.

[6] Government of Canada. Canadian Social Survey: Loneliness in Canada. November 24, 2021. Accessed August 15, 2024. https://www150.statcan.gc.ca/n1/daily-quotidien/211124/dq211124e-eng.htm.

[7] National Seniors Council. Report on the Social Isolation of Seniors. Government of Canada; 2016. Accessed August 15, 2024. https://www.canada.ca/en/national-seniors-council/programs/publications-reports/2014/social-isolation-seniors/page05.html.

[8] Penninkilampi R, Casey AN, Singh MF, Brodaty H. The association between social engagement, loneliness, and risk of dementia: a systematic review and meta-analysis. J Alzheimers Dis. 2018;66(4):1619–1633. DOI: 10.3233/JAD-180439 30452410

[9] *Social Isolation and Loneliness in Older Adults: Opportunities for the Health Care System*. National Academies Press; 2020. DOI: 10.17226/25663 32510896

[10] Fleisch Marcus A, Illescas AH, Hohl BC, Llanos AAM. Relationships between social isolation, neighborhood poverty, and cancer mortality in a population-based study of US adults. Buchowski M, editor. PLOS ONE. 2017;12(3):e0173370. DOI: 10.1371/journal.pone.0173370 28273125 PMC5342244

[11] Holt-Lunstad J, Smith TB, Baker M, Harris T, Stephenson D. Loneliness and social isolation as risk factors for mortality: a meta-analytic review. Perspect Psychol Sci. 2015;10(2):227–237. DOI: 10.1177/1745691614568352 25910392

[12] Manemann SM, Chamberlain AM, Roger VL, et al. Perceived social isolation and outcomes in patients with heart failure. J Am Heart Assoc. 2018;7(11):e008069. DOI: 10.1161/JAHA.117.008069 29794038 PMC6015354

[13] Keefe J, Andrew M, Fancey P, Hall M. A Profile of Social Isolation in Canada; 2006. https://www.health.gov.bc.ca/library/publications/year/2006/keefe_social_isolation_final_report_may_2006.pdf.

[14] Government of Canada. Population estimates, July 1, by census division, 2021 boundaries. May 22, 2024. Accessed November 25, 2024. https://www150.statcan.gc.ca/t1/tbl1/en/tv.action?pid=1710015201.

[15] Lambton Public Health. Lambton Public Health Strategic Plan 2014–2019. https://lambtonpublichealth.ca/report/2014-2019-strategic-plan/.

[16] Age-Friendly Communities Outreach Program. Creating a More Inclusive Ontario: Age-Friendly Community Planning Guide for Municipalities and Community Organizations. Published online 2021. Accessed November 28, 2024. https://files.ontario.ca/msaa-age-friendly-community-planning-guide-municipalities-community-organizations-en-2021-01-01.pdf.

[17] Age-Friendly Sarnia Steering Committee. Age-Friendly Sarnia Community Action Plan.; 2017. Accessed November 29, 2024. https://agefriendlysarnialambton.ca/Uploads/ContentDocuments/age-friendly_action_plan_final%20(11).pdf.

[18] Public Health Agency of Canada. Age-Friendly Rural and Remote Communities: A Guide.; 2014. Accessed November 29, 2024. https://www.phac-aspc.gc.ca/seniors-aines/alt-formats/pdf/publications/public/healthy-sante/age_friendly_rural/AFRRC_en.pdf.

[19] Thang LL, Yui Y, Wakabayashi Y, Miyazawa H. Promoting age-friendly community of support and care in Japan’s aging neighborhood: The Nagayama model. Aging Health Res. 2023;3(1):100111. DOI: 10.1016/j.ahr.2022.100111

[20] Yi YM, Park YH, Cho B, et al. Development of a community-based integrated service model of health and social care for older adults living alone. Int J Environ Res Public Health. 2021;18(2):825. DOI: 10.3390/ijerph18020825 33478027 PMC7835935

[21] U.S. Department of Health and Human Services Centers for Disease Control and Prevention. Office of the Director, Office of Strategy and Innovation. Introduction to program evaluation for public health programs: A self-study guide. Published online October 2011. Accessed November 25, 2024. https://stacks.cdc.gov/view/cdc/26245.

[22] Neergaard MA, Olesen F, Andersen RS, Sondergaard J. Qualitative description – the poor cousin of health research? BMC Med Res Methodol. 2009;9(1):52. DOI: 10.1186/1471-2288-9-52 19607668 PMC2717117

[23] Sandelowski M. What’s in a name? Qualitative description revisited. Res Nurs Health. 2010;33(1):77–84. DOI: 10.1002/nur.20362 20014004

[24] Canadian Institutes of Health Research, Natural Sciences and Engineering Research Council of Canada, Social Sciences and Humanities Research Council of Canada. Tri-Council Policy Statement: Ethical Conduct for Research Involving Humans.; 2018. Accessed March 7, 2022. http://publications.gc.ca/collections/collection_2019/irsc-cihr/RR4-2-2019-eng.pdf.

[25] Garcia L, Hunter R, Alsarrani A. National Programmes for Age-Friendly Cities and Communities: A Guide. World Health Organization; 2023. https://www.who.int/publications/i/item/9789240068698.

[26] Menec V, Brown C. Facilitators and barriers to becoming age-friendly: A Review. J Aging Soc Policy. 2022;34(2):175–197. DOI: 10.1080/08959420.2018.1528116 30321112

[27] Hong A, Welch-Stockton J, Kim JY, Canham SL, Greer V, Sorweid M. Age-friendly community interventions for health and social outcomes: A Scoping Review. Int J Environ Res Public Health. 2023;20(3):2554. DOI: 10.3390/ijerph20032554 36767920 PMC9915867

[28] Forsyth A, Lyu Y. Making communities age-friendly: lessons from implemented programs. J Plan Lit. 2024;39(1):3–24. DOI: 10.1177/08854122231160796

[29] Ahgren B, Axelsson R. Evaluating integrated health care: a model for measurement. Int J Integr Care. 2005;5(3). DOI: 10.5334/ijic.134 PMC1395513 16773158

[30] Valentijn PP, Schepman SM, Opheij W, Bruijnzeels MA. Understanding integrated care: a comprehensive conceptual framework based on the integrative functions of primary care. Int J Integr Care. 2013;13(1). DOI: 10.5334/ijic.886 PMC3653278 23687482

[31] Menec VH, Novek S, Veselyuk D, McArthur J. Lessons learned from a Canadian province-wide age-friendly initiative: the age-friendly Manitoba initiative. J Aging Soc Policy. 2014;26(1–2):33–51. DOI: 10.1080/08959420.2014.854606 24224864

